# Copper Tantalate by a Sodium‐Driven Flux‐Mediated Synthesis for Photoelectrochemical CO_2_ Reduction

**DOI:** 10.1002/smtd.202401432

**Published:** 2025-01-15

**Authors:** Ariadne Köche, Kootak Hong, Sehun Seo, Finn Babbe, Hyeongyu Gim, Keon‐Han Kim, Hojoong Choi, Yoonsung Jung, Inhyeok Oh, Gnanavel Vaidhyanathan Krishnamurthy, Michael Störmer, Sanghan Lee, Tae‐Hoon Kim, Alexis T. Bell, Sherdil Khan, Carolin M. Sutter‐Fella, Francesca M. Toma

**Affiliations:** ^1^ Liquid Sunlight Alliance Lawrence Berkeley National Laboratory 1 Cyclotron Rd Berkeley CA 94720 United States; ^2^ Postgraduate Program in Materials Science Universidade Federal do Rio Grande do Sul Av. Bento Gonçalves 9500 Porto Alegre RS 91540‐000 Brazil; ^3^ Department of Materials Science and Engineering Chonnam National University Gwangju 61186 Republic of Korea; ^4^ Chemical Sciences Division Lawrence Berkeley National Laboratory 1 Cyclotron Rd Berkeley CA 94720 United States; ^5^ Institute of Functional Materials for Sustainability Helmholtz‐Zentrum Hereon Kantstraße 55 14513 Teltow Germany; ^6^ Department of Chemical and Biomolecular Engineering University of California Berkeley Berkeley CA 94720 United States; ^7^ School of Materials Science and Engineering Gwangju Institute of Science and Technology Gwangju 61005 Republic of Korea; ^8^ Molecular Foundry Division Lawrence Berkeley National Laboratory 1 Cyclotron Rd Berkeley CA 94720 United States

**Keywords:** copper tantalate, flux‐mediated synthesis, photoelectrochemical CO_2_ reduction

## Abstract

Copper‐tantalate, Cu_2_Ta_4_O_11_ (CTO), shows significant promise as an efficient photocathode for multi‐carbon compounds (C_2+_) production through photoelectrochemical (PEC) CO_2_ reduction, owing to its suitable energy bands and catalytic surface. However, synthesizing CTO poses a significant challenge due to its metastable nature and thermal instability. In this study, this challenge is addressed by employing a flux‐mediated synthesis technique using a sodium‐based flux to create sodium‐doped CTO (Na‐CTO) thin films, providing enhanced nucleation and stabilization for the CTO phase. To evaluate the PEC performance and catalytic properties of the films, copper(II) oxide (CuO) at the Na‐CTO surface is selectively etched. The etched Na‐CTO shows a lower dark current, with decreased contribution from photocorrosion, unlike the non‐etched Na‐CTO which has remaining CuO on the surface. Furthermore, Na‐CTO exhibits 7.3‐fold ethylene selectivity over hydrogen, thus highlighting its promising potential as a photocathode for C_2+_ production through PEC CO_2_ reduction.

## Graphical abstract

This study presents the synthesis of metastable Cu_2_Ta_4_O_11_ via a sodium flux‐mediated approach, leading to Na‐doped Cu_2_Ta_4_O_11_ thin films. These films are employed in photoelectrochemical CO_2_ reduction, highlighting their effectiveness in producing multiple hydrocarbons.

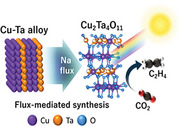

## 1 Introduction

Advancements in artificial photosynthesis for generating solar fuels are needed to effectively capture and store solar energy and push this approach to a higher technological readiness.^[^ ^1^, ^2^, ^3^, ^4^ ^]^ In this respect, photoelectrochemical (PEC) carbon dioxide reduction (CO_2_R), where carbon‐based fuels are synthesized on the illuminated surface of a semiconductor electrode using CO_2_ and solar energy, is a representative application of artificial photosynthesis designed to recycle CO_2_ into valuable fuels.^[^ ^5^ ^,^ ^6^ ^]^

Copper (Cu)‐based oxides are robust candidates for efficient PEC semiconductor electrodes, providing tunable bandgap energies ranging from 1.2 to more than 3 eV and favorable band alignments concerning redox couples for CO_2_R.^[^ ^5^, ^7^, ^8^ ^]^ Furthermore, Cu‐based oxides hold promise not only for the production of multi‐carbon hydrocarbons (C_2+_) due to the high CO binding energy at Cu sites, which promotes increased C─C coupling,^[^ ^5^, ^7^, ^9^, ^10^, ^11^ ^]^ but also for practical application based on their economic viability, earth abundance, and non‐toxic nature.

Nevertheless, Cu‐based oxides exhibit a drawback that limits their performance in PEC CO_2_R — the material corrosion is particularly pronounced in electrolytes containing OH^‐^ under illumination.^[^ ^7^, ^12^ ^]^ For instance, Cu(I) oxide (Cu_2_O) undergoes self‐reduction in an aqueous solution under irradiation due to redox potentials for oxidation and reduction lying within the bandgap, and an unsuitable electrolyte can exacerbate this degradation.^[^ ^8^, ^12^, ^13^, ^14^, ^15^ ^]^ To address this limitation, the introduction of transition metals such as niobium, tantalum (Ta), and vanadium to form ternary Cu‐based oxides has been attempted as a strategy for stabilization of the Cu(I) cation within the lattice.^[^ ^13^, ^16^, ^17^ ^]^ The promoted alteration in the coordination environment of the Cu(I) cation, coupled with the inhibition of the transfer of Cu 3*d* ^10^ valence band electrons to Cu 4*s* conduction band, proves effective in hindering Cu self‐reduction.^[^ ^13^, ^18^ ^]^ Furthermore, incorporating these elements allows for tuning the optoelectronic properties and bandgap energy within the range of 1.2 to >3 eV.^[^ ^8^ ^]^ In this respect, Cu‐based ternary oxides can be considered promising candidates with high photocathodic currents for PEC systems.^[^ ^19^ ^]^

Notably, p‐type Cu(I) tantalates, such as Cu_2_Ta_4_O_11_ (CTO), exhibit outstanding potential for PEC applications due to their redox position of band energies — composed mainly of Cu 3*d* ^10^ and Ta 5*d* ^0^ orbitals — in relation to water splitting and CO_2_R half‐reactions.^[^ ^8^, ^19^, ^20^ ^]^ However, synthesizing CTO presents a significant challenge due to its thermodynamic instability as a metastable phase.^[^ ^21^ ^]^ Hence, introducing kinetic control of the material formation is required during the synthetic process, e.g. via lower temperatures.^[^ ^17^, ^22^, ^23^ ^]^ In addition, the CTO structure has been calculated to have 33% vacancies on the Cu‐site positions within the unit cell.^[^ ^20^ ^]^ The presence of Cu vacancies induces the decomposition of CTO due to the high mobility of Cu(I) cations in the structure.^[^ ^17^ ^]^ For this reason, CTO has been mainly reported as a side product, with low reproducibility using high‐temperature (in the range of 800 to 1100 °C) synthesis.^[^ ^23^, ^24^ ^]^ Consequently, instead of relying on conventional solid‐state methods, widely used for the synthesis of metal oxides,^[^ ^16^ ^]^ alternative approaches are warranted to prepare metastable oxides like CTO.^[^ ^21^, ^22^, ^24^ ^]^

In this respect, flux‐mediated synthesis emerges as an amenable approach for the fabrication of CTO thin films. This method facilitates synthesis at comparatively lower temperatures than solid‐state synthesis, as the flux serves as a medium enhancing reactant diffusion and reaction at reduced temperatures.^[^ ^25^ ^]^ That is, selecting a suitable flux is crucial for the effective synthesis of CTO. Interestingly, the introduction of sodium (Na) ions can tune the nucleation and stabilization of the CTO phase.^[^ ^26^, ^27^ ^]^ Thus, the flux‐mediated synthesis has proven to be not only effective for the synthesis of metastable ternary metal oxide nanoparticles, providing kinetic stabilization, but also particularly crucial for incorporating the Na ion accompanied by the oxidation of metal thin films at relatively lower temperatures.^[^ ^25^ ^]^ Na^+^ presents a larger ionic radii compared to Cu^+^ and Ta^5+^, serving to expand the host lattice. This expansion results in an increase in interlayer spacing, thereby hindering Cu^+^ diffusion. Thus, Na^+^ as a dopant possesses a strong potential to improve the structural stability of CTO.^[^ ^28^, ^29^ ^]^ In this study, we successfully fabricated polycrystalline Na‐doped CTO (Na‐CTO) thin films via flux‐mediated synthesis using Na‐based flux. Through X‐ray diffraction (XRD) analysis, we confirmed the polycrystalline structure of CTO and identified the origin of secondary phases depending on the Cu, Ta, and Na ratios. For an in‐depth assessment of pristine Na‐CTO, we removed copper (II) oxide (CuO) — a frequent impurity in ternary Cu‐based oxides — from its surface and subsequently evaluated its PEC performance in CO_2_R. Notably, the synthesized Na‐CTO demonstrated a superior C_2+_ yield ratio over hydrogen (H_2_) generation in the PEC CO_2_R.

## 2 Result and Discussion

**Figure 1:**
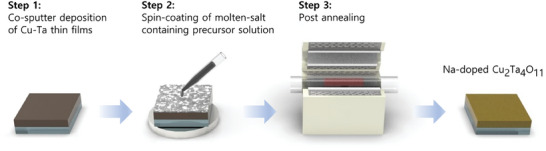
Schematic representation of the flux‐mediated synthesis procedure to obtain the Na‐CTO thin films.

The CTO thin films were synthesized by adding a solution with the salt precursors onto a metallic Cu‐Ta alloy. **Figure** **1** illustrates the procedure for the flux‐mediated synthesis. First, a co‐sputtering deposition process was employed to fabricate Cu‐Ta alloy films. Subsequently, the respective precursor solutions were spin‐coated onto the Cu‐Ta alloy films, followed by annealing. One control sample underwent annealing without any coating.

**Figure 2:**
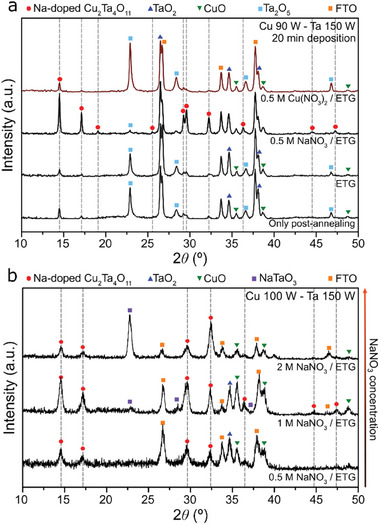
a) XRD patterns of synthesized Cu‐Ta based thin films using different flux solutions with 0.5 m Cu(NO_3_)_2_/ETG, 0.5 m NaNO_3_/ETG, and bare ETG (radiofrequency power: Cu 90 W‐Ta 150 W). b) XRD patterns of Cu‐Ta based thin films varying the concentration of the NaNO_3_ in solution (Cu 100 W‐Ta 150 W). In both diffractograms, dashed lines represent JCPDS #81‐815 pattern.

To confirm the chemical transformation and CTO phase crystallization by the flux‐mediated synthesis with Na doping, we used sodium nitrate (NaNO_3_) and copper nitrate (Cu(NO_3_)_2_) as molten salts; melting points are 308 and 115 °C, respectively.^[^ ^30^ ^]^ We prepared stock solutions of NaNO_3_ in ethylene glycol (ETG) and Cu(NO_3_)_2_ in ETG and applied them, as well as bare ETG, as precursors for the synthesis of CTO. Interestingly, the Cu‐Ta film coated with NaNO_3_ solution exhibited mainly more pronounced CTO diffraction peaks (JCPDS #81‐815) than with other precursor solutions (**Figure** **2**a).^[^ ^31^ ^]^ In contrast, the other prepared films predominantly exhibited mixed secondary phases corresponding to Ta‐based oxides and Cu‐based oxide (JCPDS #43‐1046, #89‐2843, and #48‐1548, respectively), according to Figure 2, with a minor presence of the CTO phase. To further investigate whether there is any influence of Cu‐containing salts as a flux on the synthesis of CTO or not, we applied different Cu‐containing solutions — namely copper chloride (CuCl_2_) on Cu‐Ta film, Cu(NO_3_)_2_ in ETG on both Ta and TaO_x_ films, and copper acetate (Cu(OAc)_2_) on Ta film (Figure S1, Supporting Information). These Cu flux‐based thin films exhibited secondary phases without predominant CTO peaks, suggesting that the Cu‐based flux does not promote stabilization of the CTO lattice. Collectively, the Na‐based flux is a key step in promoting the formation of the CTO phase during the flux‐mediated synthesis.

For a more comprehensive investigation into the influence of Na in the nucleation and stabilization of CTO formation, we synthesized Na‐CTO thin films using varying concentrations of NaNO_3_ (Figure 2b). When employing 0.5 m NaNO_3_, we could observe the diffraction pattern of the CTO phase along with a minor presence of Ta oxides and Cu oxides phases. Subsequently, a notable increase in the intensity of the CTO peak was observed using 1 m NaNO_3_ (Figure 2b). Further increasing of the Na content to 2 m led to the formation of NaTaO_3_ (JCPDS #73‐878).^[^ ^32^, ^33^ ^]^ Since CTO has thermodynamically intrinsic Cu(I)‐vacancies, incorporating Na into the CTO lattice can contribute to stabilizing the structure, enabling the energy delivered from calcination to enhance the crystallite size.^[^ ^26^ ^]^ However, excessive Na may lead to additional secondary phases.

To determine the origin of secondary phases, such as Cu‐based or Ta‐based oxides, we fabricated Na‐CTO polycrystalline thin films with different Ta/Cu ratios (Figure S2, Supporting Information). The Ta/Cu ratios strongly correlated with the acquisition of the CTO phase and secondary phases (Figure S2, Supporting Information). With an increase in Cu ratio, there was a noticeable increment in the proportion of the CTO phase, as well as an increase in the CuO phase. Conversely, higher Ta/Cu ratios resulted in the formation of NaTaO_3_ and Ta_2_O_5_, owing to an excess of Ta. Despite phase differences in Na‐CTO thin films depending on the Ta/Cu ratio, all Na‐CTO films show CuO diffraction peaks (Figure S2, Supporting Information). The absorption spectra of Na‐CTO films obtained with different Ta/Cu ratios were largely similar, with a slight increment in absorption with increasing Cu content due to CuO formation (Figure S3, Supporting Information). Interestingly, CuO can be formed at the surface or interface between film and substrate in Cu‐based ternary oxides.^[^ ^13^, ^19^, ^21^, ^34^ ^]^ The bulk Cu(I) ions tend to diffuse and oxidize at the surface in Cu‐based ternary oxides exposed to thermal treatment in the air atmosphere, leading to phase segregation and an increased bulk Cu(I) vacancy content.^[^ ^13^, ^19^, ^21^, ^34^ ^]^ Furthermore, CuO can be formed at the interface between film and substrate depending on the Cu ratio in Cu‐based ternary oxides.^[^ ^35^ ^]^

**Figure 3:**
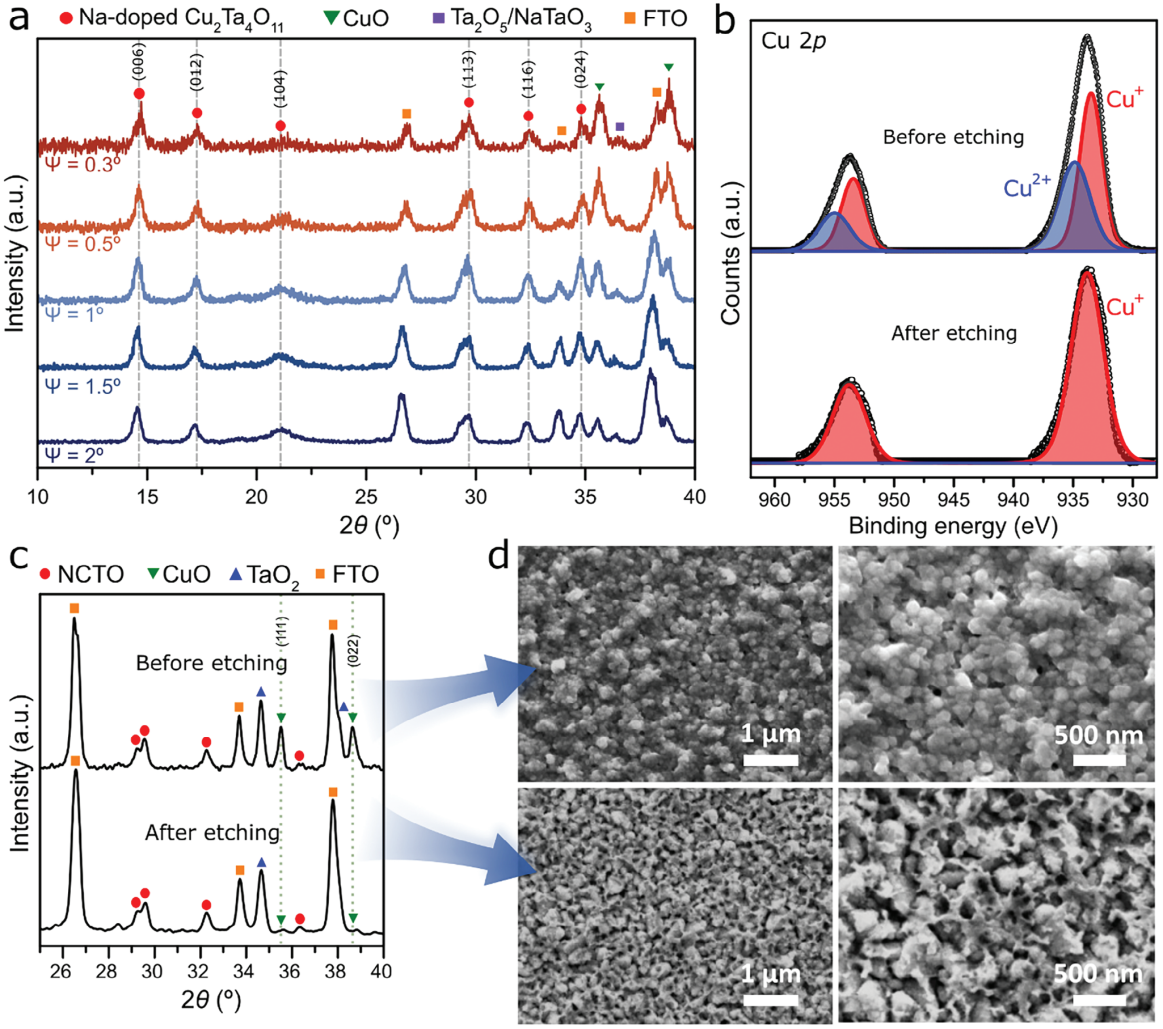
a) GIXRD patterns of Na‐CTO (Cu 100 W‐Ta 150 W) using 0.5 m NaNO_3_. Phi (Ψ) represents the grazing angle. b) High‐resolution XPS of Cu 2p region, c) XRD patterns, and d) SEM images of Na‐CTO (Cu 100 W‐Ta 150 W) thin films before and after etching with a 1 m HCl solution.

To investigate the origin of the CuO phase in the Na‐CTO thin film, we employed grazing‐incidence XRD (GIXRD). With decreasing incident angle from 2 to 0.3°, the CuO phase became more pronounced (**Figure** **3**a), indicating that CuO was formed at the surface of the Na‐CTO films. Although surface CuO on the Na‐CTO films can enhance photocurrent generation and promote charge separation,^[^ ^19^ ^]^ it also renders the photoelectrode susceptible to photo corrosion.^[^ ^5^, ^15^ ^]^ To mitigate this susceptibility, we eliminated the surface CuO phase using a 1 m HCl solution. After this chemical etching process, the Cu^2+^ species previously detected through X‐ray photoelectron spectroscopy (XPS) analysis present on the Na‐CTO surface were no longer detected (Figure 3b),^[^ ^36^, ^37^ ^]^ with only Cu^+^ species originating from the Na‐CTO remaining.^[^ ^12^ ^]^ Also, the peaks corresponding to CuO (111) and (022) planes significantly diminished in the XRD characterization (Figure 3c). The remaining slight CuO peaks detected may arise from residual CuO originating at the interface between Na‐CTO and fluorine‐doped tin oxide (FTO) substrate. To confirm the presence of CuO at the interface, we employed transmission electron microscopy (TEM) and energy‐dispersive X‐ray spectroscopy (EDS) analysis for the Na‐CTO films (Figure S4, Supporting Information). The CuO was formed not only at the surface but also at the interface between Na‐CTO and FTO substrate, as shown in Figure S4 (Supporting Information). Additionally, after etching, the morphology of the obtained films displayed cavities and a rough surface due to the removal of CuO from the surface in comparison to before the etching process, as depicted in the scanning electron microscopy (SEM) images in Figure 3d.

**Figure 4:**
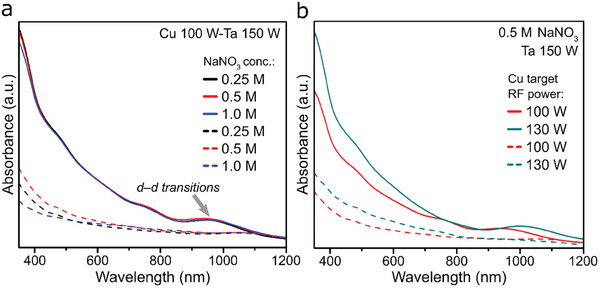
Absorbance spectra of a) Na‐CTO (Cu 100 W‐Ta 150 W) thin films with different NaNO_3_ concentrations, and b) Na‐CTO thin films produced with different Ta/Cu ratios, showing the results before etching (solid lines) and after etching (dashed lines) for both graphs.

Furthermore, we conducted (UV–vis) measurements for etched and non‐etched Na‐CTO thin films to compare differences of light absorption properties. These films exhibited distinct broad absorption patterns in the visible light region (**Figure** **4**). A minor band, situated in the near‐infrared region (900–1000 nm), is also noticeable, primarily attributed to *d–d* transitions of Cu^2+^ originating from CuO.^[^ ^38^ ^]^ Interestingly, altering the concentration of the Na precursor yielded identical absorption spectra, whereas this absorption is significantly diminished after the removal of the CuO from the film surface through etching, in comparison to the spectra observed before etching (Figure 4a). The diminished band ≈1000 nm is a consequence of the filled 3*d* ^10^ configuration of the Cu^+^ species in the Na‐CTO phase, leading to low expected absorption from them.^[^ ^38^ ^]^ A similar behavior is observed in absorbance spectra shown in Figure 4b, comparing CTO films with different Ta/Cu ratios before and after etching. As anticipated, increased Cu deposition results in a broader absorption profile for the thin films, accompanied by a slight red‐shift in the spectra, due to a greater content of the higher‐energy 3*d* ^10^ orbitals.^[^ ^20^ ^]^

**Figure 5:**
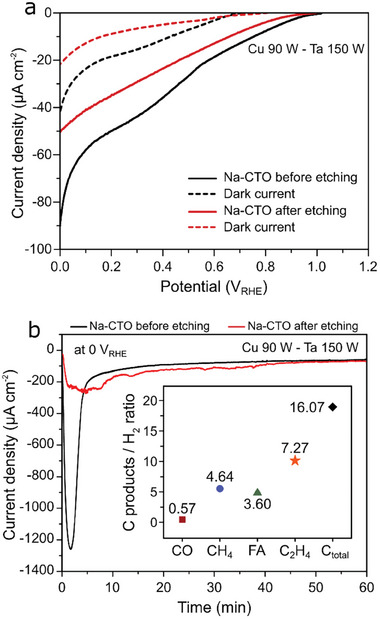
a) LSV curves were obtained for the Na‐CTO (Cu 90 W‐Ta 150 W) with different NaNO_3_ concentrations as salt precursors, showing the results for the films before etching (solid lines) and after etching (dashed lines) with a 1 m HCl solution. b) CA measurements for Na‐CTO (Cu 90 W‐Ta 150 W) for the films before and after etching (inset: C products/H_2_ of etched Na‐CTO thin films depicting different products from CO_2_R).

To ascertain the viability of Na‐CTO thin films as a prospective candidate for PEC photoelectrodes in CO_2_R, we measured PEC performances for the Na‐CTO films, both with and without etching, employing a three‐electrode configuration in a CO_2_ saturated 0.1 m KHCO_3_ electrolyte solution under AM 1.5 G solar irradiation conditions with Ag/AgCl reference electrode and Pt counter electrode. In linear sweep voltammetry (LSV) measurement, the non‐etched Na‐CTO thin film exhibited a notably higher dark current density of ≈−42 µA cm^−2^ and a photocurrent density of −90 µA cm^−2^ at 0 V versus reversible hydrogen electrode (RHE) (**Figure** **5**a), which can be attributed to the presence of surface CuO on non‐etched Na‐CTO thin film.^[^ ^39^ ^]^ Conversely, the etched Na‐CTO thin film demonstrated a comparatively diminished dark current density of ≈−22 µA cm^−2^ at 0 V_RHE_, indicating that the corrosion of the photoelectrode is significantly reduced.^[^ ^40^ ^]^

To corroborate the occurrence of CuO corrosion during CO_2_R measurements, we conducted chronoamperometry (CA) measurements at 0 V_RHE_ over 1 h, as shown in Figure 5b. The non‐etched Na‐CTO thin films exhibited an abrupt increase and then decrease in current within the first 4 min, which can be attributed to the combined effects of CuO photocorrosion and the photogenerated charge carriers by CuO.^[^ ^7^, ^19^, ^40^, ^41^, ^42^ ^]^ However, this initial corrosion peak was significantly reduced in the etched film, thus supporting the proposal that Na‐CTO is relatively stable in comparison to CuO, thus removing surface CuO from the obtained films can enhance the overall stability of the photoelectrode.

Additionally, we performed XRD measurements to compare the non‐etched Na‐CTO (Cu 130 W‐Ta 150 W sample) before and after PEC measurements. Our analysis revealed that when the samples exhibit peaks corresponding to secondary phases such as CuO and Ta_2_O_5_, the PEC measurement is significantly diminished. After PEC measurements, the sample before etching shows the appearance of Ta (100) and (110) peaks at ≈12° and 38.5°, as well as a Cu (111) peak ≈43°, while the CTO phase remained stable during the PEC analysis (Figure S5, Supporting Information). This observation further reinforces the conclusion that the CTO phase is more stable compared to the secondary phases under CO₂ reduction conditions.

For further verification of the photoelectrode while minimizing the effects of corrosion, we conducted Incident Photon‐to‐Current Efficiency (IPCE) measurements at 0.4 V_RHE_, a potential where the corrosion of CuO is relatively less pronounced (Figure S6, Supporting Information). Both etched and non‐etched Na‐CTOs exhibited comparable current density and efficiency overall. However, the etched Na‐CTO demonstrated higher efficiency in the range of 450–500 nm, thus indicating more effective charge transfer. Since the efficiency measured by IPCE excludes the influence of dark current, this finding suggests that the etched CTO sample more effectively utilizes the current generated by light for CO_2_R.

Furthermore, we analyzed hydrocarbons (C) products/H_2_ ratio for the etched Na‐CTO using the calculated Faradaic efficiency (*FE*) based on CA result to validate its effectiveness in producing value‐added carbon products from CO_2_R (inset of Figure 5b). The etched Na‐CTO produced various carbon‐based products, including ethylene (C_2_H_4_), methane (CH_4_), formic acid (FA), and carbon monoxide (CO); The FE of each product was included in Table S1 (Supporting Information). Specifically, the C total/H_2_ ratio was determined to be 16, and the ratio of C_2_H_4_ to H_2_ was found to be 7.3.

Production of C_2+_ gas products, particularly ethylene, has not been demonstrated in many other PEC systems. For example, organometal halide perovskite‐based photocathodes have yet to report ethylene production,^[^ ^43^, ^44^, ^45^ ^]^ and while Cu₂O have shown promise, they do not produce ethylene in aqueous electrolytes.^[^ ^12^ ^]^ Silicon‐based photocathodes are capable of ethylene production but still exhibit high overpotentials.^[^ ^46^, ^47^ ^]^ In contrast, the FE of CTO shows the remarkable selectivity of Cu(I) ternary oxides in facilitating CO_2_R, even at 0 V_RHE_.^[^ ^7^, ^11^ ^]^

However, the etched Na‐CTO also exhibited degradation in photocurrent during CA measurements. Therefore, additional strategies should be considered to enhance charge separation and augment photocurrent generation. One potential approach for improvement could involve the introduction of a charge transport layer^[^ ^40^, ^48^ ^]^ to engineer the band structure or passivation techniques,^[^ ^40^, ^49^ ^]^ among others. Additionally, since Na doping can reduce carrier concentration due to its different oxidation states, it would be beneficial to identify an element that can simultaneously stabilize the CTO phase while increasing the carrier concentration. These approaches may contribute to further optimizing the performance of etched Na‐CTO as a photoelectrode for efficient CO_2_R.

## 3 Conclusion

Na‐CTO films were synthesized successfully using a flux‐mediated synthesis approach, offering a valuable method for the stabilization of the CTO phase at mild temperatures, with the presence of Na in the reaction medium. The elimination of the CuO phase from the surface resulted in a noticeable reduction in dark current without severe corrosion, indicating relatively enhanced stability essential for PEC applications. Significantly, the elevated C_2_H_4_/H_2_ ratio, surpassing 7.3 times, highlights the promising potential of Na‐CTO as a photocathode for the efficient generation of C_2+_ products in PEC CO_2_R. This investigation propels future research, advocating the exploration of Cu(I)‐based ternary oxides as viable, cost‐effective, earth‐abundant, and non‐toxic alternatives for photoelectrodes in the realm of CO_2_R.

## 4 Experimental Section

### Preparation of Na‐CTO Thin Films

For the synthesis of Na‐CTO thin films, no unexpected or unusually high safety hazards were encountered. The chemicals were used without further purification. FTO substrates (Sigma–Aldrich, 100 mm x 100 mm x 2 mm, TEC 15, with nominal resistivity of ≈13 Ω) were cleaned with methanol and isopropyl alcohol prior to deposition. Cu‐Ta thin films were deposited onto a fluorine‐doped tin oxide (FTO) coated glass substrate through radiofrequency (RF) magnetron co‐sputtering (AJA International, Inc.) at 10 mTorr background pressure under an argon atmosphere, with varying deposition times ranging from 15 to 40 min. To control the Ta/Cu ratio, different RF powers were applied to the Cu target (70–210 W), while the power to the Ta target was kept at 150 W. Sputtering targets were purchased from Kurt J. Lesker. For comparison, Ta only and TaO_x_ films were also prepared on FTO substrate. The latter was obtained by thermal treatment of deposited Ta film in an air atmosphere at 600 °C. Before precursorsalts coating, a 3 min O_2_ plasma treatment was conducted on the films to create a hydrophilic surface. Subsequently, solutions of Cu‐ or Na‐containing salts were spin‐coated (Laurell Technologie) onto the films (3000 r.p.m., 60 s, with an acceleration rate of 150 r.p.m. s^−1^ for first 3 s). Namely, 0.25–2 m sodium nitrate (NaNO_3_, Sigma–Aldrich, ≥99.0%) was used in ETG (Sigma–Aldrich, 99.8%), 0.5 m copper(II) nitrate (Cu(NO_3_)_2_, Sigma–Aldrich, ≥99.9%) in ETG, copper(II) chloride (CuCl_2_, Sigma–Aldrich, 99%), and bare ETG on the Cu/Ta alloy films, as well as Cu(NO_3_)_2_ in ETG on a TaO_x_ film, and copper(II) acetate (Cu(OAc)_2_, Sigma–Aldrich, 98%) on Ta film. The films coated with the precursor salts were dried on a hot plate at 100 °C for 5 min. The spin‐coating process was carried out a total of two times. Following this, the coated thin films underwent annealing at 600 °C for 3 or 10 h under air using a box furnace (Cole‐Parmer). As a control sample, bare Cu‐Ta thin films were also annealed simultaneously under the same conditions.

### Characterization

The crystalline structure of the films was analyzed by XRD (SmartLab, Rigaku) using Cu Kα radiation (λ = 0.15 418 nm) with a 0.045° step size and measurement time of 0.4 s per step. GIXRD was used with a grazing angle of 0.3–2° range. Obtained XRD data was indexed using the International Centre for Diffraction Data (ICDD) – PDF2 database. XPS (Kratos) analysis was performed with a monochromatic Al Kα line (ℎν = 1486.6 eV). Spectral fitting was conducted using the Casa XPS analysis software. The C 1s peak of adventitious carbon was fixed at 284.8 eV to set the binding energy scale. The absorption spectra of Na‐CTO thin films on FTO substrates were measured using a UV–vis spectrophotometer (SolidSpec‐3700, Shimadzu). The morphological and structural characterization of the thin films was carried out using SEM (Quanta FEG 250, FEI) with an acceleration voltage of 10 kV. For TEM analysis, specimens were prepared by conventional methods of mechanical polishing followed by Ar ion milling. TEM images were obtained with a 200 kV field‐emission TEM (FE‐TEM, JEM‐2100F, JEOL). The EDS (X‐MaxN 80T, Oxford instruments) installed in the FE‐TEM was used to investigate the chemical element mapping of the Na‐CTO thin films.

### PEC Measurements

Assessments of the PEC performance of the obtained thin films were conducted by employing a three‐electrode configuration using a Pt foil counter electrode and leak‐free Ag/AgCl (saturated KCl) reference electrode under an 0.1 m KHCO_3_ (99.995%, Sigma–Aldrich) electrolyte solution pumped and cycled into the system using a peristaltic pump (Thermo Scientific). The area of the photoelectrode was 1 cm^2^ and PEC tests were conducted under simulated AM 1.5 G sunlight at 100 mW cm^−2^. A homemade PEC cell was used for PEC test.^[^ ^50^ ^]^ The CO_2_ flow rate was controlled by a mass flow controller (Alicat Scientific) and was kept constant at 5 sccm. LSV was measured at a scan rate of 20 mV s^−1^ from open circuit potential to 0 V_RHE_ using a potentiostat (Biologics). Chronoamperometry was measured at 0 V_RHE_. The IPCE measurements were conducted using a monochromatic light source and a set of optical filters to adjust the wavelength. The measurements covered a wavelength range of 300–625 nm with intervals of 5 nm. Both the etched and non‐etched Na‐CTOs were evaluated under identical conditions to compare their PEC performance. All potentials have been converted to RHE scale according to the following equation:

(1) $$E_{\mathrm{RHE}}=E_{\mathrm{Ag}/\mathrm{AgCI}}+0.197+0.0591\times \mathrm{pH}$$

where E_Ag/AgCl_ was the potential relative to the Ag/AgCl electrode.

The evolved gaseous products were detected and quantified with an inline gas chromatograph (GC, Agilent Technologies 7890B) coupled with a thermal conductive detector and helium ionization detector. The FE associated with the product to calculate the ratio of carbon products to H_2_ (C products/H_2_) was determined using the following equation:

(2) $$FE=\frac{nFcV}{I}\times 100\%$$

where *n* represents the number of electrons transferred, *F* denotes Faraday's constant, *c* was the molar concentration of the species, *V* represents the total volumetric flow rate, and *I* was the measured total current.

No unexpected or unusually high safety hazards were encountered.

## Conflict of Interest

The authors declare no conflict of interest.

## Supporting information



Supporting Information

## Data Availability

The data that support the findings of this study are openly available in [Zenodo] at [https://doi.org/10.5281/zenodo.12703344], reference number [12703344].
