# Supplementary material for: Copper Tantalate by a Sodium‐Driven Flux‐Mediated Synthesis for Photoelectrochemical CO2 Reduction
Source: Small Methods. 2025 Jan 15;9(8):2401432. doi: 10.1002/smtd.202401432 (PMC12391617; doi:10.1002/smtd.202401432)
Supplement: Supplementary file 1 — Supporting Information [file SMTD-9-2401432-s001.docx]

Supporting Information

Copper tantalate by a Sodium-driven flux-mediated synthesis for photoelectrochemical CO_2_ reduction

Ariadne Köche, Kootak Hong, Sehun Seo,^*^ Finn Babbe, Hyeongyu Gim, Keon-Han Kim, Hojoong Choi, Yoonsung Jung, Inhyeok Oh, Gnanavel Vaidhyanathan Krishnamurthy, Michael Störmer, Sanghan Lee, Tae-Hoon Kim, Alexis T. Bell, Sherdil Khan, Carolin M. Sutter-Fella,^*^ Francesca M. Toma^*^

^A. Köche, S. Seo, F. Babbe, K.-H. Kim, A. T. Bell, F. M. Toma^

^Liquid Sunlight Alliance^

^Lawrence Berkeley National Laboratory^

^1 Cyclotron Rd, Berkeley, CA 94720, United States^

^A. Köche, S. Khan^

^Postgraduate Program in Materials Science^

^Universidade Federal do Rio Grande do Sul^

^Av. Bento Gonçalves 9500, Porto Alegre - RS, 91540-000, Brazil^

^K. Hong, H. Gim, T.-H. Kim^

^Department of Materials Science and Engineering^

^Chonnam National University^

^Gwangju, 61186, Republic of Korea^

^S. Seo, F. Babbe, K.-H. Kim, A. T. Bell, F. M. Toma^

^Chemical Science Division^

^Lawrence Berkeley National Laboratory^

^1 Cyclotron Rd, Berkeley, CA 94720, United States^

^S. Seo, H. Choi, G. V. Krishnamurthy, M. Störmer, F. M. Toma^

^Institute of Functional Materials for Sustainability^

^Helmholtz-Zentrum Hereon^

^Kantstraße 55, 14513 Teltow, Germany^

^K.-H. Kim, A. T. Bell^

^Department of Chemical and Biomolecular Engineering^

^University of California Berkeley^

^Berkeley, CA 94720, United States^

^Y. Jung, I. Oh, S. Lee^

^School of Materials Science and Engineering^

^Gwangju Institute of Science and Technology^

^Gwangju, 61005, Republic of Korea^

^C. M. Sutter-Fella^

^Molecular Foundry Division^

^Lawrence Berkeley National Laboratory^

*^1 Cyclotron Rd, Berkeley, CA 94720, United States^*


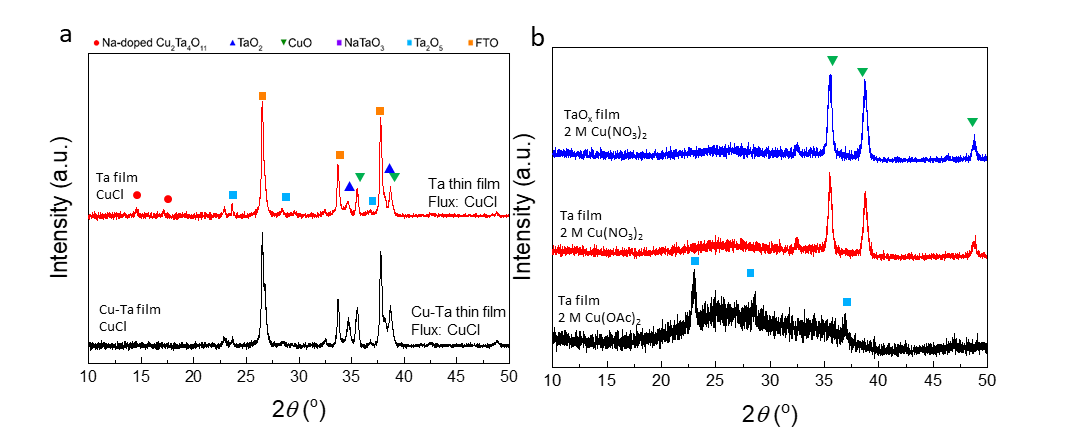


**Figure S1.** X-ray diffraction (XRD) patterns of synthesized copper (Cu)/tantalum (Ta)-based thin films using different Cu solutions as precursors. (a) Copper(II) chloride (CuCl_2_)-employed Cu-Ta and Ta thin films on fluorine-doped tin oxide (FTO) substrates. After spin-coated with CuCl_2_, the films were annealed at 600 ^o^C for 12 h. (b) Copper nitrate (Cu(NO_3_)_2_)-employed TaO_x_ and Ta thin film and copper(II) acetate (Cu(OAc)_2_)-employed Ta thin film on silicon substrates. These films were annealed at 600 ^o^C for 3 h.


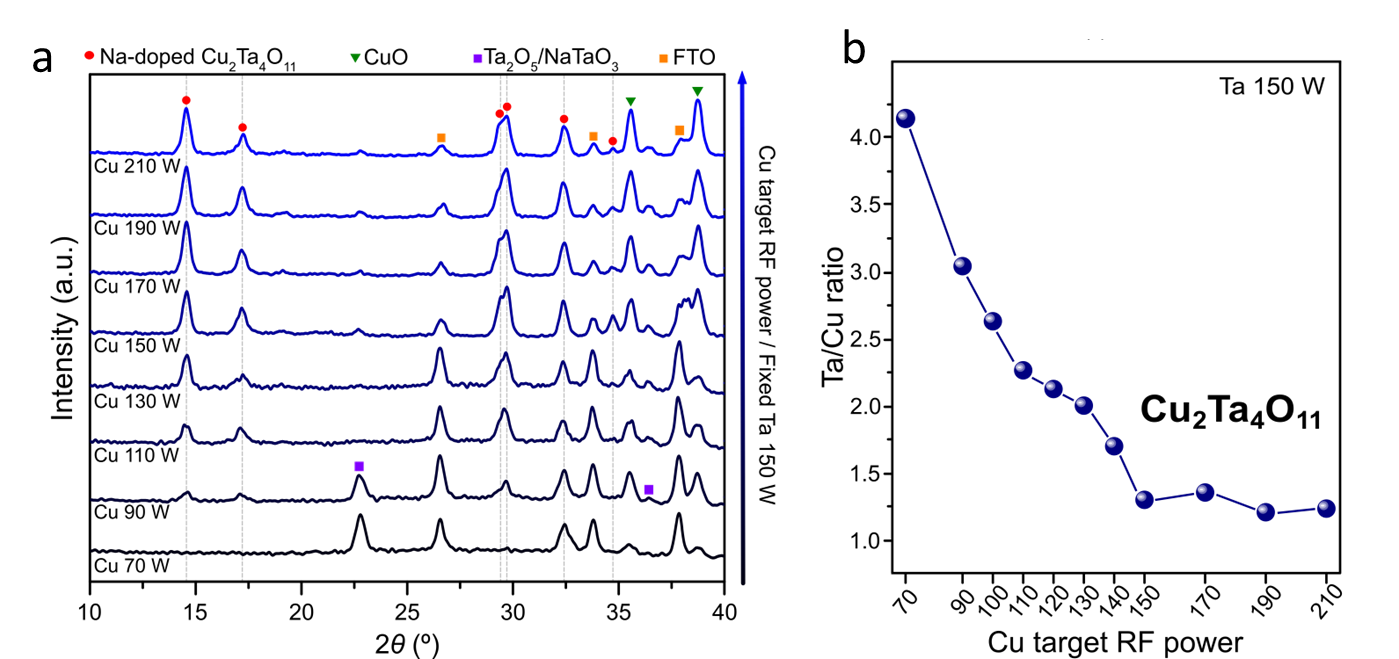


**Figure S2.** (a) XRD patterns of obtained samples with increasing Cu target radiofrequency (RF) power and its effect in the sodium (Na)-doped CTO (Na-CTO) phase formation. (b) Ta/Cu ratio depending on the different Cu target RF power through energy dispersive spectroscopy measurement.


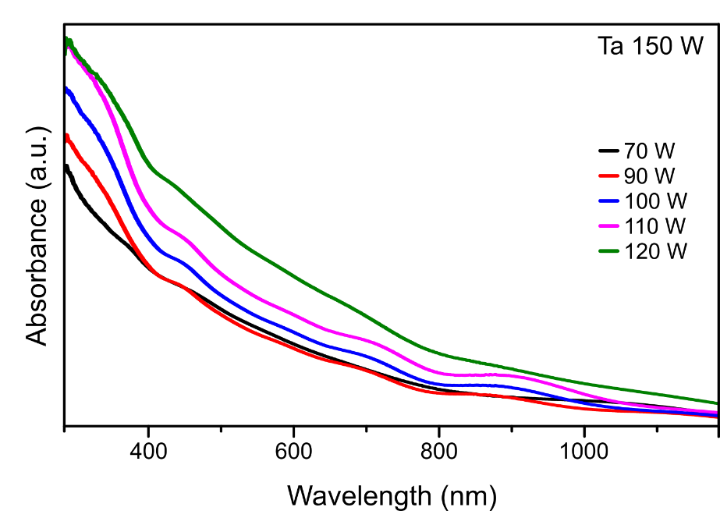


**Figure S3.** Absorbance spectra of Na-CTO samples obtained with different Ta/Cu ratios by changing Cu target RF power during sputtering deposition while keeping Ta target RF power constant at 150 W and using 1 M sodium nitrate (NaNO_3_) in ethylene glycol (ETG) solution for spin-coating.


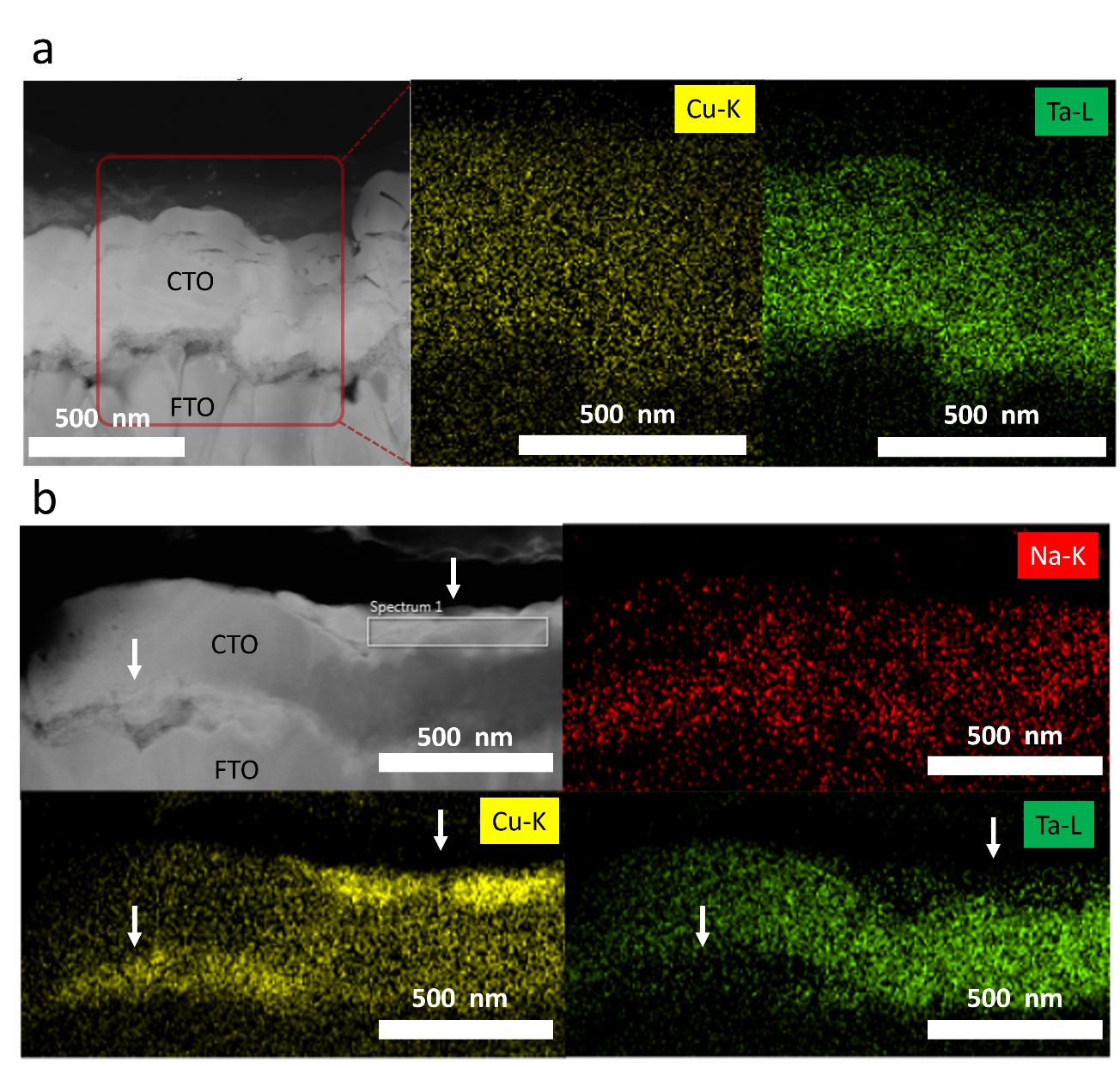


**Figure S4.** Transmission electron microscopy (TEM) and energy-dispersive X-ray spectroscopy (EDS) images of a Na-CTO thin film (Cu 90 W/ Ta 150 W, 20 min). (a) CTO phases are predominantly observed on the FTO substrate. The Cu-K EDS map reveals a relatively widespread distribution of Cu over the CTO surface compared to the Ta-L EDS map, suggesting the potential formation of copper(II) oxide (CuO) on the CTO. (b) The formation of CuO (indicated by a white arrow) in specific CTO regions, both on the CTO surface and at the interface between the CTO and the FTO substrate. The Na-K EDS map demonstrates effective doping and uniform distribution of Na throughout the entire thin film.


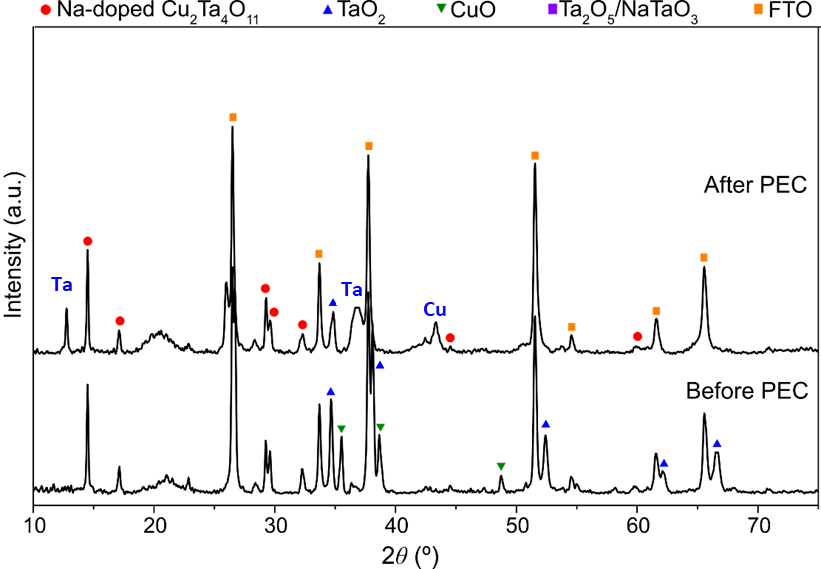


**Figure S5**. XRD patterns of Na- CTO thin films obtained using 130 W for Cu target RF power and 150 W for Ta target RF power during sputtering deposition, and 0.5 M NaNO_3_ in ETG solution for spin-coating, before and after PEC performance tests (linear sweep voltammetry and Chronoamperometry measurements).

**Figure S6**. IPCE for both etched and non-etched Na-CTO across the wavelength range of 300–625 nm.

**Table S1**. Faradaic efficiency (*FE*) of Na-CTO with etching.

|  | **H_2_** | **CO** | **FA** | **Methane** | **Ethylene** | **Total** | **C product** |
| --- | --- | --- | --- | --- | --- | --- | --- |
| Na-CTO with etching (%) | 1.91 | 1.08 | 8.87 | 6.88 | 13.90 | 32.64 | 30.73 |
